# Early pregnancy overweight/obesity and length of residence among immigrants in Sweden: a pooled analysis of Swedish population registers between 1992 and 2012

**DOI:** 10.1017/S1368980020004231

**Published:** 2021-04

**Authors:** Dilana López-Borbón, Jesper Löve, Sol P Juárez

**Affiliations:** 1 School of Public Health and Community Medicine, University of Gothenburg, Gothenburg, Sweden; 2 School of Nursing, University of Costa Rica, San José, Costa Rica; 3 Centre for Health Equity Studies (CHESS), Stockholm University/Karolinska Institutet, Stockholm, Sweden; 4 Department of Public Health Sciences, Stockholm University, SE-106 91 Stockholm, Sweden

**Keywords:** Country of origin, Global health, Inequalities, Length of residence, Migration, Overweight/obesity, Pregnancy

## Abstract

**Objective:**

To examine whether the association between women’s origin and early pregnancy overweight and obesity (OW/OB) varies by length of residence in Sweden.

**Design:**

This cross-sectional observational study used pooled Swedish population register data from 1992 to 2012. Logistic regression models were run to estimate odds ratios (OR) of early pregnancy OW/OB and 95 % confidence intervals (95 % CI), comparing Swedish-born and immigrants by length-of-residence categories while adjusting for covariates.

**Setting:**

Sweden.

**Participants:**

In total, 1 771 821 pregnant women, 315 992 of whom were immigrants.

**Results:**

With longer residence in Sweden, more immigrant women from various origins exhibited higher odds of experiencing early OW/OB compared with Swedish-born women. Findings specifically showed increased odds of early pregnancy OW/OB with increasing length of residence for women born in Latin America, Europe-27 and Southeast Asia. For example, immigrant women from Latin America residing in Sweden for < 6 years showed similar odds as Swedish-born (OR_≤ 5 years_ 0·92, 95 % CI 0·87, 0·98), while their longer residing counterparts showed higher odds than Swedish-born women (OR_6–15 years_1·21, 95 % CI 1·14, 1·28 and OR_≥ 16 years_ 1·68, 95 % CI 1·59, 1·78). Mixed results were found for other origins.

**Conclusions:**

The current study suggests that host country conditions might play an important role in explaining OW/OB among some groups of immigrant women. Although further studies are needed to disentangle the mechanisms that generate these health inequalities, policy efforts should focus on immigrant reception and early integration to prevent pregnancy-related OW/OB.

Overweight and obesity (OW/OB) is increasing worldwide, particularly within vulnerable populations such as immigrants^(1,2)^. In Sweden, around 50 % of adults are OW/OB^(3)^. Early pregnancy OW/OB among women has particularly increased in recent decades (from ~25 % in 1992 to ~40 % in 2018)^(4)^. Early pregnancy OW/OB is not only associated with increased risk of gestational diabetes, hypertension, preeclampsia and depression in the mother^(5–7)^ but also with adverse outcomes for the child at birth (e.g. preterm birth or death)^(8,9)^ and across the life span (e.g. adult obesity)^(5,10)^. Given the public health implications of early pregnancy OW/OB for the mother and child alike, it is necessary to identify instruments that can help monitor changes in the population. For immigrants specifically, information on length of residence can illustrate these changes while revealing the extent to which conditions in the host country can amplify, reduce (modify) or maintain the initial risks.

Studies have shown that immigrants’ health deteriorates with increasing length of residence^(11,12)^ including through increased obesity^(13,14)^. However, few studies have specifically focused on pregnant women using total population registers or have only scrutinised immigrants from origins predominantly representing labour migration. These specificities could largely limit the generalisability of the findings.

The aim of the current study is to fill in the above-mentioned knowledge gaps by examining the prevalence of early pregnancy OW/OB by the length of residence among migrant women living in Sweden in order to evaluate whether longer time in Sweden is associated with a higher prevalence of OW/OB. Sweden is unique in the sense that it has a long history of receiving both labour and asylum-seeking immigrants from all continents. This, together with the high quality of its population registers, represents a unique context in which to investigate this topic.

## Data and methods

### Data

This is a cross-sectional population-based register study. The data for the current study were retrieved from the Swedish Medical Birth Register and the Longitudinal Integration Database for Health Insurance and Labour Market Studies, which were nominally linked by Swedish authorities, covering the period between 1992 and 2012. Additionally, the Population Register was used to retrieve information on the country of origin and year of arrival in Sweden. The study population consists of 1 771 821 women who gave birth in Sweden, of which 315 992 were immigrants and 1 455 829 were Swedish-born.

### Exposure variables

Country/region of birth was the main exposure variable, categorised into: Sweden (reference); EU-27 (Europe-27 excluding Sweden); Eastern Europe (mainly from Russia, former Yugoslavia); Middle East (mainly from Iraq, Lebanon, Iran and Turkey); Afghanistan; Ethiopia; Eritrea; rest of Africa; Southeast Asia; South Asia; Latin America (predominantly from Chile); Oceania (mainly New Zealand and Australia) and North America (Canada and the USA). This categorisation was selected after considering the prevalence of OW/OB across countries of origin by length of residence. The main grouping criterion was geographical proximity, unless patterns of OW/OB by length of residence differed within a region, in which case countries were included separately (e.g. Eritrea and Ethiopia with respect to the rest of Africa) or grouped together if samples were too small (i.e. Oceania and North America). Length of residence was categorised into three groups (≤ 5 years, 6–15 years and ≥ 16 years). In order to examine OW/OB among immigrants by length of residence while using the Swedish-born population as a reference, a combined variable using country/region of origin and length of residence was created.

### Outcome variable and covariates

Early-pregnancy OW/OB was the outcome variable. It was estimated from the weight and height reported to or measured by midwives during the first antenatal visit (between 8 and 12 weeks of gestation)^(15)^ and dichotomised as OW/OB (BMI ≥ 25 kg/m^2^) and not OW/OB (<25 kg/m^2^), based on international recommendations^(16)^.

Other variables considered in the current study were age (≤ 24, 25–29, 30–34 and ≥ 35 years); education (low: ≤ 9 years; intermediate: 10–13 years; high: ≥ 14 years); family situation (cohabiting with the father or single/other) due to its connection with emotional and economic support; employment (yes/no); parity (1, 2, 3 or ≥ 4 children); current smoking (non-smokers; 1 to 9 cigarettes per day; 10 or more cigarettes per day) and household disposable income (quintiles) including salary, parental leave corresponding to a previous child (if any) or any other benefits estimated for the year before the birth.

### Statistical analysis

Multivariable logistic regression models were run in order to assess the association between women’s country/region of origin, length of residence and OW/OB during early pregnancy. Odds ratios (OR) and 95 % confidence intervals (95 % CI) were presented. Models were adjusted for calendar year, age, family situation, parity, employment, education, disposable income and current smoking. A sensitivity analysis was conducted to assess the impact of missing information in the BMI variable (7·8 %) based on the strong assumption that all missing observations were OW/OB. The results of the sensitivity analyses were almost identical to the main findings. All models included robust SE to account for the existence of siblings.

All analyses were performed using the SPSS Statistics software package, version 25 (SPSS Inc.).

## Results

Table 1 summarises the characteristics of the study population. Compared to Swedish-born women, immigrant women were more likely to be unemployed (56·2 % *v*. 17·6 %) to have a low income (44·6 % *v*. 13·5 %) and a low level of education (32·3 % *v*. 9·9 %). However, immigrant women smoked less (9·4 % *v*. 11·5 %), though this prevalence increased with longer residence.

**Table 1:** Sociodemographic characteristics for women giving birth by regions/countries of origin and length of residence in Sweden 1992–2012 (*n* 1 771 821)

			Immigrants by country of origin and length of residence in Sweden		
	Sweden	Immigrants	≤ 5 years	6–15 years	≥ 16 years
	*n* 1 455 829 %	*n* 315 992 %	*n* 141 579 %	*n* 104 423 %	*n* 69 990 %
Region/country of origin					
EU-27		24·3	20·0	22·1	36·2
Eastern Europe		18·2	17·8	20·8	15·2
Middle East		24·2	28·8	25·4	13·3
Afghanistan		1·0	1·4	1·1	0·2
Eritrea		1·0	1·1	1·2	0·4
Ethiopia		1·8	1·4	2·5	1·5
Rest of Africa		9·9	12·9	10·3	3·2
South-East Asia		9·3	9·5	7·7	11·3
South Asia		2·8	1·9	1·7	6·5
Oceania and North America		1·3	1·3	1·3	1·5
Latin America		6·1	4·0	5·9	10·8
Age at childbearing					
≤ 24	33·3	30·6	33·5	26·9	30·2
25–29	15·5	19·5	26·0	14·6	13·5
30–34	33·7	29·8	26·3	33·5	31·4
≥ 35	17·5	20·2	14·2	25·0	24·9
Family situation					
Cohabitant	95·3	91·8	93·4	89·8	91·7
Single/other	4·7	8·2	6·6	10·2	8·3
Parity					
1	43·6	37·1	44·7	27·4	36·3
2	37·5	35·1	35·7	34·7	34·4
3	13·8	16·6	12·3	21·9	17·3
≥ 4	5·1	11·2	7·3	16·0	11·9
Education (years)					
Low (≤ 9)	9·9	32·3	40·4	31·8	16·6
Intermediate (10–13)	49·0	34·8	24·8	37·9	50·2
High (≥14)	41·1	33·0	34·8	30·3	33·2
Employment					
Yes	82·4	43·8	22·3	53·6	72·6
No	17·6	56·2	77·7	46·4	27·4
Household disposable Income (quintiles)					
1 (Lowest)	13·5	44·6	55·4	42·6	25·6
2	19·9	22·1	21·5	22·8	22·3
3	22·1	12·8	9·2	13·9	18·4
4	22·7	10·1	6·5	10·5	17·0
5 (Highest)	21·9	10·4	7·4	10·3	16·6
Current smoking (Cigarette/d)					
No	88·4	90·6	93·2	90·4	85·5
1–9	7·9	6·7	5·1	6·9	9·7
≥10	3·6	2·7	1·7	2·6	4·8

All values were statistically significant considering a two-sided *P* value < 0·05, using Sweden-born as a reference group.

Table 2 displays the prevalence of early pregnancy OW/OB for Swedish-born and immigrant women by country/region of origin and length of residence. Compared with the prevalence of OW/OB among Swedish-born women (33 %), most immigrant groups had either lower or similar prevalence upon arrival (between 14 % for Southeast Asia and 33 % for ‘Oceania and North America’), with the exception of the ‘rest of Africa’, whose prevalence was higher (49 %). Yet, with each increasing category of length of residence, a larger number of immigrant countries/regions of origin exhibited an OW/OB prevalence exceeding that of natives. Among immigrants residing in Sweden for 6 to 15 years, only three immigrant groups displayed a lower prevalence (EU-27, Eritrea and South-East Asia at 31 %, 33 % and 18 %, respectively), down to only one group among those who had been in Sweden for more than 15 years (Southeast Asia at 24 %).

**Table 2:** Prevalence of early pregnancy OW/OB (> 25 kg/m^2^) by regions/countries of origin and length of residence in Sweden 1992–2012 (*n* 1 771 821)

		Length of residence in Sweden (among migrants)		
	Sweden	≤ 5	6–15	≥ 16
	*n* 1 455 829	*n* 141 579	*n* 104 423	*n* 69 990
Region/country of origin	% OW/OB	% OW/OB	% OW/OB	% OW/OB
Sweden	33·4			
EU-27		26·4	31·1	**37·6**
Eastern Europe		30·8	**38·3**	**37·8**
Middle East		**42·2**	**50·0**	**42·7**
Afghanistan		32·4	**47·5**	**49·5**
Eritrea		26·2	32·5	**37·7**
Ethiopia		32·7	**38·1**	**40·7**
Rest of Africa		**48·8**	**60·2**	**52·2**
South- East Asia		14·0	18·4	24·1
South Asia		28·0	**38·1**	**34·4**
Oceania and North America		32·9	**35·4**	32·8
Latin America		**33·4**	**41·4**	**50·0**

The bold number highlights the region/country that has a higher prevalence of OW/OB compared with the reference group. All values were statistically significant considering a two-sided *P* value < 0·05, using Sweden-born as a reference group.

Table 3 shows the results of comparing early pregnancy OW/OB between Swedish-born and immigrant women grouped by country/region of origin and length of residence. Results showed that almost all immigrant group with < 6 years of residence in Sweden showed similar or lower odds of experiencing early pregnancy OW/OB than Swedish-born women (with OR ranging from 0·26; 95 % CI 0·24, 0·27 for Southeast Asia to 0·82; 95 % CI 0·73, 0·92 for Ethiopia). The exceptions were for women coming from the ‘rest of Africa’ and the Middle East, who showed higher odds (OR_≤ 5 years_ 1·42 95 % CI 1·37, 1·48; and OR_≤ 5 years_ 1·19 95 % CI 1·16, 1·22, respectively). Immigrant women from more countries/regions of origin showed greater odds of early pregnancy OW/OB compared with Swedish-born women when residing in Sweden for 6 to 15 years, including those from the ‘rest of Africa’ (OR 2·02 95 % CI 1·93, 2·13), Middle East (OR 1·47 95 % CI 1·43, 1·51), Latin America (OR 1·21 95 % CI 1·14, 1·28), Afghanistan (OR 1·20 95 % CI 1·04, 1·37) and ‘Oceania and North America’ (OR 1·21 95 % CI 1·06, 1·39), all of which also showed increased odds compared with their more recently arrived counterparts (those in category of residence ≤ 5). This pattern extended to immigrant groups residing in Sweden for 16 years or longer, including immigrants from Latin America (OR 1·68 95 % CI 1·59, 1·78), the ‘rest of Africa’ (OR 1·61 95 % CI 1·46, 1·78), Middle East (1·16 95 % CI 1·10, 1·22) and EU-27 (OR 1·10 95 % CI 1·07, 1·14). Other groups also had increased but non-significant odds, including Afghanistan (OR 1·39 95 % CI 0·92, 2·10) possibly due to their small sample sizes.

**Table 3:** Multivariable association of combined variable of women’s regions/countries of origin and length of residence and OW/OB (> 25 kg/m^2^) *v*. No OW/OB in Sweden 1992–2012 (*n* 1 771 821)

	≤ 5 *n* 141 579		6–15 *n* 104 423		≥ 16 *n* 69 990	
	OR	95 % CI	OR	95 % CI	OR	95 % CI
Sweden (ref)	1		1		1	
EU-27	0·70	0·68, 0·73	0·87	0·84, 0·90	**1·10**	**1·07, 1·14**
Eastern Europe	0·72	0·70, 0·74	0·93	0·90, 0·96	0·95	0·91, 1·00
Middle East	**1·19**	**1·16, 1·22**	**1·47**	**1·43, 1·51**	**1·16**	**1·10, 1·22**
Afghanistan	0·68	0·61, 0·76	**1·20**	**1·04, 1·37**	1·39	0·92, 2·10
Eritrea	0·47	0·42, 0·54	0·66	0·57, 0·76	0·83	0·62, 1·11
Ethiopia	0·82	0·73, 0·92	0·89	0·81, 0·98	1·01	0·86, 1·17
Rest of Africa	**1·42**	**1·37, 1·48**	**2·02**	**1·93, 2·13**	**1·61**	**1·46, 1·78**
South-East Asia	0·26	0·24, 0·27	0·34	0·32, 0·37	0·57	0·53, 0·61
South Asia	0·71	0·65, 0·78	0·98	0·88, 1·09	0·94	0·87, 1·01
Oceania and North America	1·09	0·98, 1·22	**1·21**	**1·06, 1·39**	0·98	0·83, 1·15
Latin America	0·92	0·87, 0·98	**1·21**	**1·14, 1·28**	**1·68**	**1·59, 1·78**

Model adjusted for calendar year, age, family situation, parity, employment, education, disposable income and current smoking. All estimates are derived from the same model. Ref = reference category; in bold are the regions/country that are statistically significant higher OR compared with Swedish women.

Immigrant women coming from Latin America and EU-27 showed increased odds of early pregnancy OW/OB with each increasing length-of-residence category, departing from similar or lower odds to higher odds than Swedish-born women (OR_≤ 5 years_ 0·92 95 % CI 0·87, 0·98; OR_6–15 years_ 1·21 95 %CI 1·14, 1·28; OR_≥ 16 years_ 1·68 95 % CI 1·59, 1·78 for Latin America; and OR_≤ 5 years_ 0·70 95 % CI 0·68, 0·73; OR_6–15 years_ 0·87 95 % CI 0·84, 0·90; OR_≥ 16 years_ 1·10 95 % CI 1·07, 1·14 for EU-27).

## Discussion

With longer residence in Sweden, migrant women from more countries/regions of origin exhibited higher odds of experiencing early pregnancy OW/OB compared to Swedish-born women. Findings specifically showed higher odds of early pregnancy OW/OB with increasing length of residence for women born in Latin America, EU-27 and Southeast Asia. Mixed results are found for other origins although, overall, most migrant origins with 6 to 15 years of residence in Sweden tended to show higher odds than their counterparts with < 6 years of residence.

The possibility that immigrants adopt unhealthy risk behaviours (including physical inactivity and poor dietary habits) leading to an increased prevalence of OW/OB is supported by previous studies^(17,18)^ and could well be explained by the general adverse social circumstances that immigrants encounter in the new country (e.g. unemployment, low economic resources and social isolation)^(19)^. In support of this, studies conducted in Sweden showed that obesity is higher among migrant women who experience economic difficulties^(18)^ as well as among those who live in deprived neighbourhoods, consisting mainly of people with low education and low socio-economic status^(20)^. Hence, we interpret our results through a health inequity lens^(21)^ and advocate for a ‘Health in All Policy Approach’ (HiAP)^(22)^ that calls for intersectoral efforts to prevent OW/OB in general and among women of reproductive age specifically. Given that OW/OB is a multifactorial health problem, the efforts should cover a wide range of welfare policies, from warranting access to antenatal care to enhancing general living conditions. However, with more studies investigating the mechanisms behind our present findings, including the relation between upstream and downstream social determinants of health, subsequent policy actions should become even more refined.

The suggested ‘downward trend’ observed in the current study for some migrant groups residing more than 15 years in Sweden (compared with their more recently migrated counterparts) has not been documented previously, and thus remain to be explained. One possibility relates to selection through out-migration (e.g. if immigrants who suffered from poorer health are more likely to return to their homeland). Future studies should therefore study these groups more specifically.

### Strengths and limitations

We used population-based register data, which are less subject to problems of selection, non-response, interviewer effect or attrition than survey methods. The long timespan of the study in combination with long migratory tradition in Sweden is one of the major strengths, as we are able to assess multiple categories of length of residence while isolating the effect of calendar year. However, there are some limitations worth mentioning. First, it is not possible to know from the register the extent to which weight is self-reported or measured by midwife, which might be a problem is substantial differences exist between migrant groups or if the quality of the information varies by length of residence. Second, a substantial portion of information on women’s weight and/or height was missing. However, our sensitivity analysis, which was based on a strong assumption of OW/OB in all missing observations, suggests that our findings are robust (*results available upon request*). Third, the length of residence information might not be precise for refugees since it may be based on the date that they were granted their residence permit rather than the date when they actually arrived in Sweden. Fourth, information on weight and height is not available for non-pregnant migrant women in Sweden at the national level, so, unfortunately, we cannot compare our results with those of the general female population.

Although we believe that length of residence could be a relevant instrument to monitor immigrants’ health, we are aware of the limitation of using cross-sectional data for this purpose, as we actually derived trends by comparing unrelated women who gave birth at different stages of their residence in Sweden. However, previous studies conducted with the same register, using roughly the same years but looking at other outcomes concluded that the results were consistent with those from longitudinal studies, i.e. by comparing the results to a subsample of women who gave birth multiple times^(21,23)^.

## Conclusions

The results of the present study suggest that the host country might play an important role in explaining varying patterns of early pregnancy OW/OB among immigrants with different lengths of residence. Although further studies are needed to confirm the results using longitudinal data and to disentangle the mechanisms involved, we advocate for intersectoral efforts to tackle this problem as early as the reception and early integration phases of residence.
